# Plasmonic Chromatic Electrode with Low Resistivity

**DOI:** 10.1038/s41598-017-15465-8

**Published:** 2017-11-09

**Authors:** Young Gyu Moon, Yun Seon Do, Min Ho Lee, Bo Yeon Hwang, Dong Jun Jeong, Byeong-Kwon Ju, Kyung Cheol Choi

**Affiliations:** 1 Korea Advanced Institute of Science and Technology (KAIST), School of Electrical Engineering, Daejeon, 34141 Republic of Korea; 2 Kyungpook National University, School of Electronics Engineering, Daegu, 41566 Republic of Korea; 3 Korea University, School of Electrical Engineering, Seoul, 136-713 Republic of Korea

## Abstract

We report on the optical and electrical properties of a novel plasmonic chromatic electrode (PCE). The PCE was composed of a metallic nano-hole array and ITO layer as a dielectric for electrical property. The structure design was optimized to obtain the matched condition between surface plasmon modes at the top and bottom metal-dielectric interfaces for high transmittance. The fabricated PCEs have high transmittance of 25~40% and low resistivity (level of 10^−5^ Ωcm) compared to conventional electrodes. Due to the multi-functionality and simple structure of PCEs, we predict the PCEs can be applied for advanced industrial use such as, high resolution, flexible, and stretchable devices.

## Introduction

The technical convergence produced by nano technologies has spurred the development of novel transparent, flexible, stretchable, and other multi-functional devices. Among the many interesting features of nano structures, they have the remarkable ability to produce colors when they interfere with visible light. A nanostructure inspired by the components of a Morpho butterfly’s wing appears vivid blue without any colored ingredient^1^. Such structurally colored materials are being increasingly introduced as an alternative to conventional color technology^2,3^. Unlike synthesized dyes or pigments^4^, a nano structure produces color based on simple design rules. In addition, structurally colored materials have higher stability when exposed to chemicals, heat or light than organic synthesized color materials.

Notably, color engineering with metal nano structures has been reported to be an excellent strategy for fabricating color filters^5–8^. Metallic nanostructures exhibit strong resonance in the visible light range, due to the collective oscillation of free electrons at the surface, in a process called surface plasmon resonance (SPR). The surface plasmons (SPs) can propagate along the metal surface (PSPs) or become localized at discontinuities (LSPs)^9,10^. Particle-shaped metal structures accompany LSPs and appears colors due to absorption and scattering at resonance wavelength^11^. Randomly distributed particles produce the SP momentum vectors in random direction. Finally it results in weak strength of resonance consequently affecting poor color selectivity.

In contrast, metallic nano-hole arrays (MNAs) combine both PSPs and LSPs simultaneously, thus the structure provide higher possibility of coupling between existing light modes. Additionally, the momentum of SPs in a periodic structure is well-arranged along the direction of the array. In this way, periodic hole structure show better filtering characteristics such as high transmission and good color selectivity. We previously reported on MNA-based chromatic filters, including their design method and integration on realistic devices;^12^ the fabrication method for large area applications;^13^ and an MNA-based plasmonic filter integrated on transparent TFTs, which had the effect of improving the instability of TFTs under light^14^.

In the same vein, here we suggest additional advanced MNAs which function as both color filter and electrode, called plasmonic chromatic electrodes (PCE). The fabricated PCE is superior to conventional transparent or colored electrodes, resulting in 35%, 41.5% and 28.1% transmission in the red, green and blue region, respectively. Furthermore, the resistivity of the red, green and blue electrodes was found to be 3.13 × 10^−5^ Ωcm, 3.67 × 10^−5^ Ωcm and 2.91 × 10^−5^ Ωcm, respectively. These PCEs also provide better color selectivity than our first proposed chromatic electrode based on LSPs^11^.

By combining the color filter and transparent electrode, the complexity of the device structure and fabrication processes can be significantly reduced. The proposed PCE is just 220 nm thick for red and green, and 145 nm for blue. This thin film device is also preferable for integration on shape-free (flexible or stretchable) electronics. We expect that these multifunctional MNA devices will open new opportunities to develop and expand the use of novel transparent and flexible nanostructures.

## Design of PCEs

When light encounters MNAs, SPs are excited at the metal-dielectric interface, SP polaritons provide additional light modes, and this results in a higher potential for coupling between the light modes of the incident light and the SP modes of the top and bottom metal-dielectric interfaces. Eventually, the level of transmission at the SPR frequency (or wavelength) exceeds the aperture ratio of the nano-holes. The enhancement is much larger than described in classical optic theory (Bethe’s theory)^15^ so this phenomenon is called extraordinary optical transmission (EOT). The exact mechanism of the EOT can be interpreted by calculating the contribution of SPR and the quasi-cylindrical wave^16,17^. However, we only consider the contribution of SPR because SPs are dominant factors on EOT in the visible range^16^. With this respect, EOT can be predicted with a simple equation which roughly computes the SPR wavelength corresponding to the spectral position of the transmittance peak with the central wavelength, λ_SP_:^10,18–22^

1 $${\lambda }_{sp}=\frac{P}{\sqrt{{m}^{2}+{n}^{2}}}\sqrt{\frac{{\varepsilon }_{m}{\varepsilon }_{d}}{{\varepsilon }_{m}+{\varepsilon }_{d}}}$$

where P is the period of the nano-hole array in metal; ε_m_ and ε_d_ denote the permittivity of the metal and dielectric materials, respectively. The resonance mode is determined by the (m,n) number which represents the reciprocal vectors in the 2D array. Generally, the transmission intensity originating from EOT exhibits maximum value at the longest λ_SP_ (or at the lowest energy level) which corresponds to (1,0) or (0,1). The second longest λ_SP_ shifts to the shorter wavelength region with 1/√2 times value from Equation 1. Therefore, we used the observed longest λ_SP_ as the central wavelength of the pass band region.

**Figure 1:**
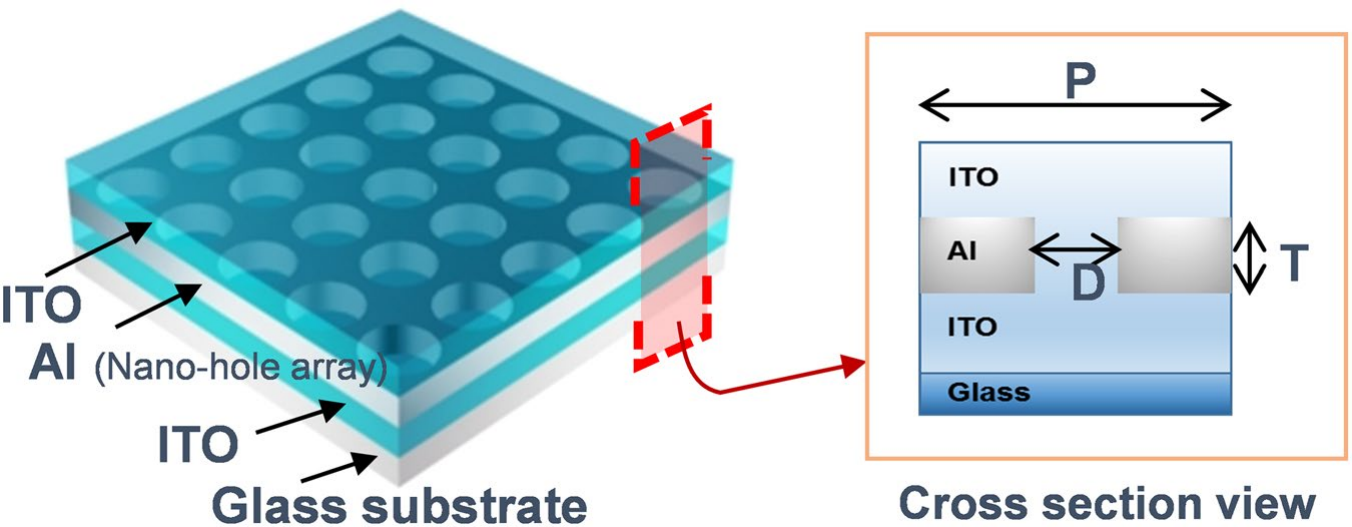
Schematic of the plasmonic chromatic electrode design.

Figure 1 illustrates the schematics of the suggested PCE. Transparent dielectric, metal, and transparent dielectric materials are stacked in turn to tens of nanometers. Nano sized perforated holes are repeated in the metal film with a period ‘P’. SPR is ignited in noble metals such as gold, silver and aluminum (Al). The interband transition of Al is away from the region of ultra violet and visible light, and that reduces the optical losses of the SPR in the visible range^23,24^. In addition, noble metals have high DC conductivity, so they also provide beneficial electric properties as an electrode. In order to realize high transmittance and low resistivity, we used indium tin oxide (ITO), one of the superior transparent conducting oxides, as the plasmonic dielectric material.

Since the MNAs have a metal-dielectric interface at the top and bottom, two different SP modes exist. When the SP modes of the top and bottom faces of the MNAs are identical, i.e., they are being generated at the same wavelength, the EOT efficiency is maximized and high transmittance is obtained. The material dependence of EOT in Equation 1 is described by the permittivity of the bulk state. However, the SPs are confined at the surface, and the strength decays exponentially along the depth direction. Consequently, we derived the effective thickness of ITO needed to create identical SP modes between the top and bottom, even with the symmetry-broken structure.

To accomplish this, we performed a numerical analysis using the finite-different time-domain (FDTD) method. The SP mode on the bottom face was determined by combining a glass substrate and a 60 nm thick ITO layer. The 60 nm thick ITO_B results in a flat morphology by our sputtering equipment. In order to enhance EOT sufficiently, the dimension of metal thickness and hole size should be less than half wavelength of light^25^. We defined as follows: the thickness of the Al film was 60 nm; the period of the MNAs was 140 nm and the diameter of the holes was 100 nm.

**Figure 2:**
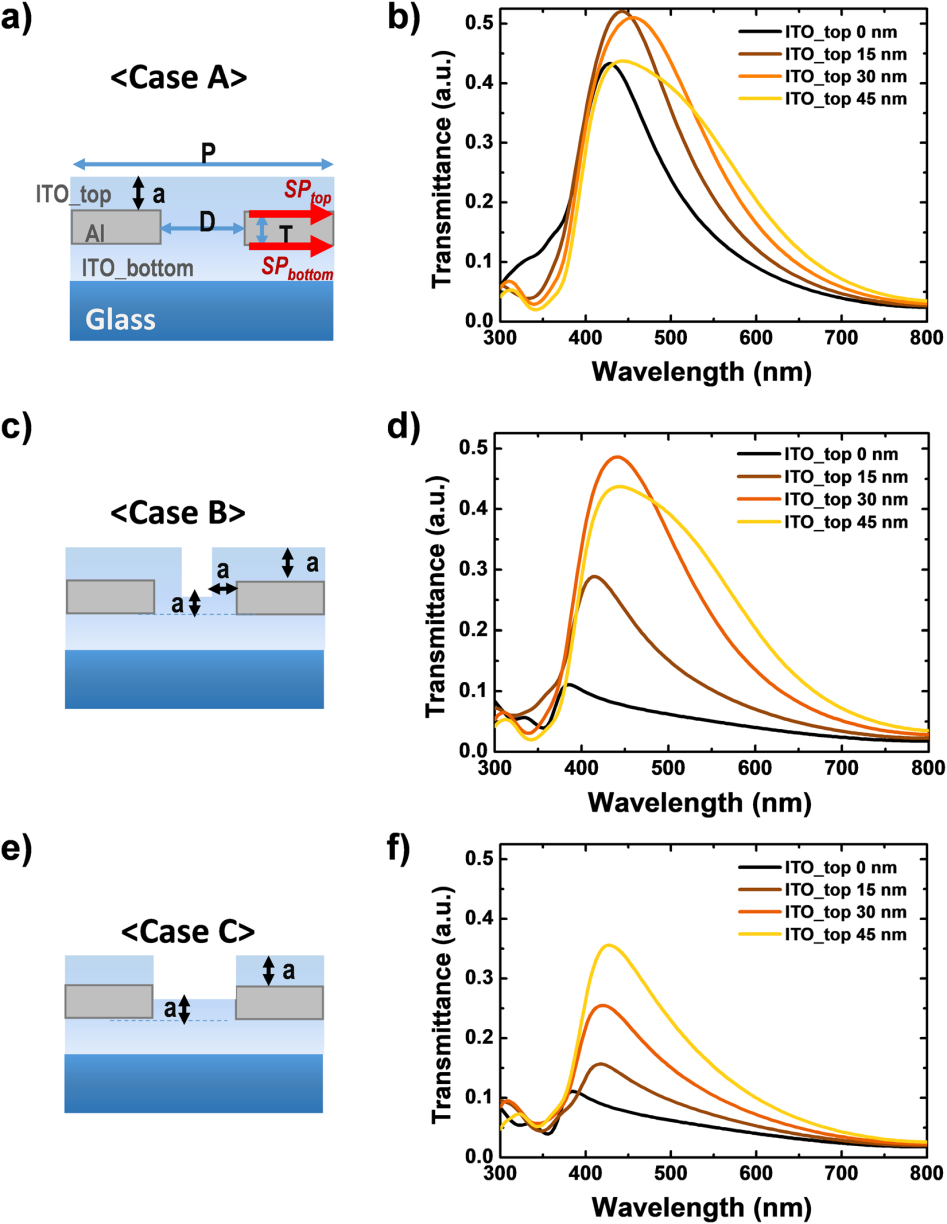
Transmission spectra of the PCEs classified by deposition types: (**a**) flat(fully-filled hole with ITO), (**c**) conformal, (**e**) poor step coverage. The graphed data (**b,d,f**) indicates transmittance according to the thickness of the top ITO layer of each case (0 nm~45 nm).

In order to generate SP modes that were matched between the top and bottom faces, we selected three different realizable structures, as shown in Fig. 2. Each case was separated by the step coverage of the area inside the hole. In Case A, the hole was fully filled with ITO, which was the same dielectric material used for both the top and bottom of the MNAs. Case B assumed there was a conformal deposition. Case C had a poor step-coverage, where the materials were only deposited in a vertical direction. Case A can be simply formed with a coating technology, but conformal deposition can also be used when the top ITO layer becomes thicker.

In practical, although the deposited ITO follows the morphology of the under surface it become smoother and quasi-flat as the thickness of deposition become larger. Usually, the larger thickness than the morphology scale of the target surface meet this situation. Additionally, since ITO is a lossy medium (Figure S1 in Supporting information) and has large refractive index, the loss of transmittance increases as the ITO_top layer become thicker. Considering these conditions, the simulation was performed for a thickness smaller than that of the Al layer (60 nm).

The graphs in Fig. 2 show the transmittance spectra according to the thickness of the top ITO layer, ‘a’, which varies from 0 nm to 45 nm. As the thickness of the top ITO layer increases up to 30 nm, all of the structures show higher transmittance. In terms of peak intensity, Case A was superior to the other structures, showing an increase from 37.4% to 52.0%. Case B and Case C resulted in changes from 9.6% to 48.6% and from 9.6% to 25.5%, respectively. In addition, the shift in λ_SP_ had the minimum value for Case A; it was 28 nm (437 nm to 465 nm) in Case A; 57 nm (394 nm to 451 nm) in Case B; 39 nm (394 nm to 430 nm) in Case C.

Interestingly, the state of the inside of the hole affects the identical SP modes on the top and bottom faces. As the top ITO layer becomes thicker, the effective refractive index (n_eff_) of the top dielectric media changes from the refractive index of air (n = 1.0) to that of ITO. The larger n_eff_ results in the peak being at a longer wavelength, as expected from Equation 1. Therefore, the peak shift and higher transmittance which occurs when the thickness of the ITO on the top is increased are important clues to the matching SP modes^13^. This trend is well described in Case B (Fig. 2d), in which all of the exposed surfaces including the cylindrical walls inside the holes are covered with the same thickness of ITO.

On the other hand, the λ_SP_ of Case A without the top ITO (a = 0 nm) was 437 nm, larger than the structures with empty holes. And the shift in λ_SP_ reached saturation at an ITO layer thickness of around 30 nm; the peak transmittance slightly decreased after that. This means the two SP modes occurred in the same wavelength region and resulted in the highest transmittance. An ITO top layer thicker than 30 nm could cause a reduction in the out-coupled light due to the lossy ITO media (Supporting information Figure S1), and 45 nm of ITO resulted in 43.7% (9.3% reduction compared to the maximum value at the spectra of ITO_top 15 nm case).

In comparison to the results of the matched SP mode in Case A, the other two cases showed different aspects. The spectrum of Case B with a 30 nm thick ITO top layer was quite similar to that of Case A with a 30 nm thick ITO top layer. The difference between the two spectra was 3.6%p in peak transmittance and 14 nm in λ_SP_. Case B needed a slightly thicker ITO top layer than Case A to generate identical SP modes.

As the thickness of ITO_top becomes larger, Case A and B showed early transmission enhancement and transmission degradation due to loss in thicker ITO. The graph of Case C shows the only trend of a red-shift in the λ_SP_ and higher transmittance with a thicker ITO top layer. However, the amount of λ_SP_ shift and the increase in peak intensity were much less than in Case A, where identical SP modes were created. In addition, when the ITO_top layer was 45 nm the transmittance was still less than other structures. The matched condition was also confirmed by the electric field profiles at λ_SP_ of each case in Supporting information. (Figure S4)

From the results in Fig. 2, we concluded that a fully filled hole was the most effective way to realize high transmittance, so we designed the optical characteristics of the PCE using Case A. Conformal deposition is an effective way of dealing with ITO. Therefore, to form a flat top surface like the one in Case A, we assumed that the thickness of the top ITO layer would be more than 50% of the Al thickness.

**Figure 3:**
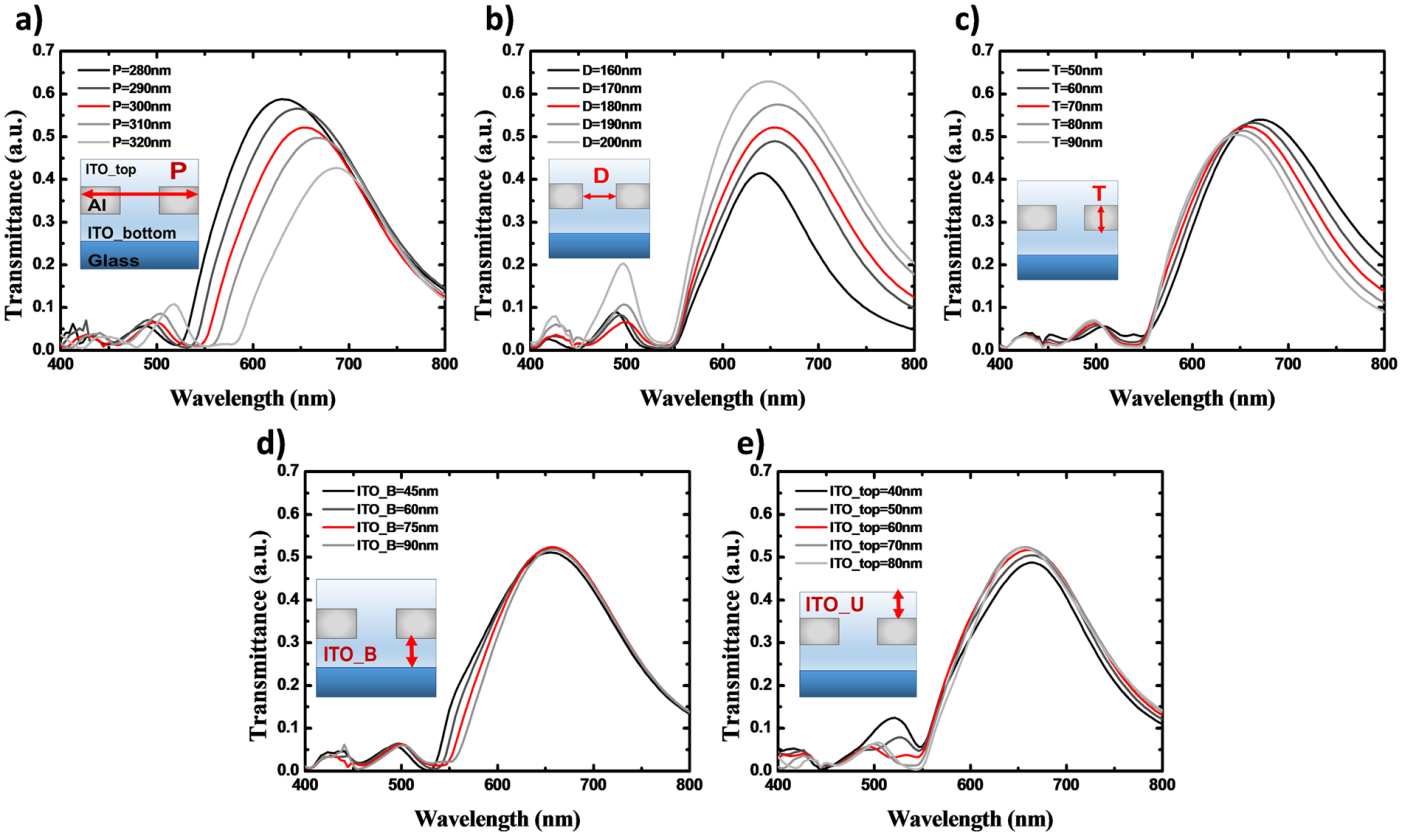
Calculated transmittance spectra of the red plasmonic chromatic electrode (PCE_R) according to (**a**) period of MNAs, P; (**b**) diameter of holes, D; (**c**) thickness of the Al film, T; (**d**) thickness of the bottom ITO layer, ITO_B; (**e**) thickness of the top ITO layer, ITO_U. The inset of each graph indicates the unit cell of the simulated area.

To adjust the target color range, we optimized the PCE structure so that the SPR would ignite in the proper wavelength region. As indicated in Equation 1, the period of the MNAs is the major factor determining SPR wavelength. The thickness of the dielectric layers on the top and bottom faces determines the efficiency of EOT by matching SP modes, as aforementioned. In addition, other detailed parameters were precisely controlled to design the spectral response of the MNAs. The design of the PCEs was performed in the order shown in Fig. 3: a) the period of the MNAs, P; b) the diameter of the holes, D; c) the thickness of the Al film, T; d) the thickness of the bottom ITO layer, ITO_B; and e) the thickness of the top ITO layer, ITO_U.

Figure 3 shows the spectra of the MNAs, which were designed to pass the color red with a peak transmittance higher than 50%, in the wavelength range from 600 nm to 700 nm. The period of the MNAs was calculated to adjust the λ_SP_. In order to determine the effect of the period by itself, the thickness of each layer was set to be 75 nm larger than the penetration of the SPs. The initial diameter was 180 nm. As shown in Fig. 3a, a larger P resulted in a longer λ_SP_ . As P was varied from 280 nm to 320 nm, the λ_SP_ shifted from 630 nm to 687 nm. When P was 300 nm the spectrum had 655 nm of λ_SP_ and the peak transmittance was 52.2%.

Using the determined P, the diameter of the holes and the thickness of the Al was investigated, as shown in Fig. 3b,c. The other dimensions were still the same as the initial values. A larger D led to higher transmittance, but the filtering band became broader and the λ_SP_ shifted. A thinner T resulted in an aspect similar to the case with larger D, but the peak shift was very small. We selected the following as the best conditions: 1) a transmittance of over 50% at 650 nm of λ_SP_; 2) the smallest full width half maximum (FWHM); 3) the lowest 2nd peak, to reduce the color mixing. The hole diameter was finalized as 180 nm, where the peak transmittance was 52.1% at 652 nm and the FWHM was 165 nm.

70 nm thick Al slightly improved the performance, resulting in a 52.4% peak transmittance and 162 nm of FWHM. Lastly, the thicknesses of ITO_B and ITO_U were derived using the highest transmittance with minimal changes. When ITO_B was less than 75 nm, the peak transmittance declined and the spectrum was broadened. 95 nm of ITO_B reduced the peak transmittance by 0.5% as compared to 75 nm of ITO_B. The maximum peak transmittance value, of 52.4% at 655 nm, was obtained when ITO_U was 75 nm.

In this way, PCEs for green and blue were designed, as described in the Supporting Information (Figures S2 and S3). Since the materials we used absorb UV and the deep blue range, the transmittance of the blue PCE was lower than the transmittance obtained with the green and red PCEs. The finalized dimensions for the red, green, and blue PCEs are summarized in Table 1. The maximum transmittances were 55.4% at 567 nm, and 33.9% at 479 nm, for the green and blue PCEs, respectively.

**Table 1:** The calculated dimensions of the optimized PCE structure.

Color	Period [nm]	Diameter [nm]	Al thickness [nm]	ITO_top [nm]	ITO_bottom [nm]
Red	300	180	70	75	75
Green	230	150	70	75	75
Blue	180	100	55	45	45

## Fabricated PCE, Optical and Electric properties

We fabricated a red PCE (PCE_R), a green PCE (PCE_G), and a blue PCE(PCE_B) and confirmed their optical and electric performances. The fabrication process used was the one previously suggested for large area application^13^. ITO and Al were deposited on a 2.5 cm × 2.5 cm area of a glass substrate by sputtering and thermal evaporation, respectively. In order to generate the nano-hole arrays on the Al layer, laser-interference-lithography (LIL) was used^26–28^. Finally, ITO was deposited again on top of the Al MNAs.

**Figure 4:**
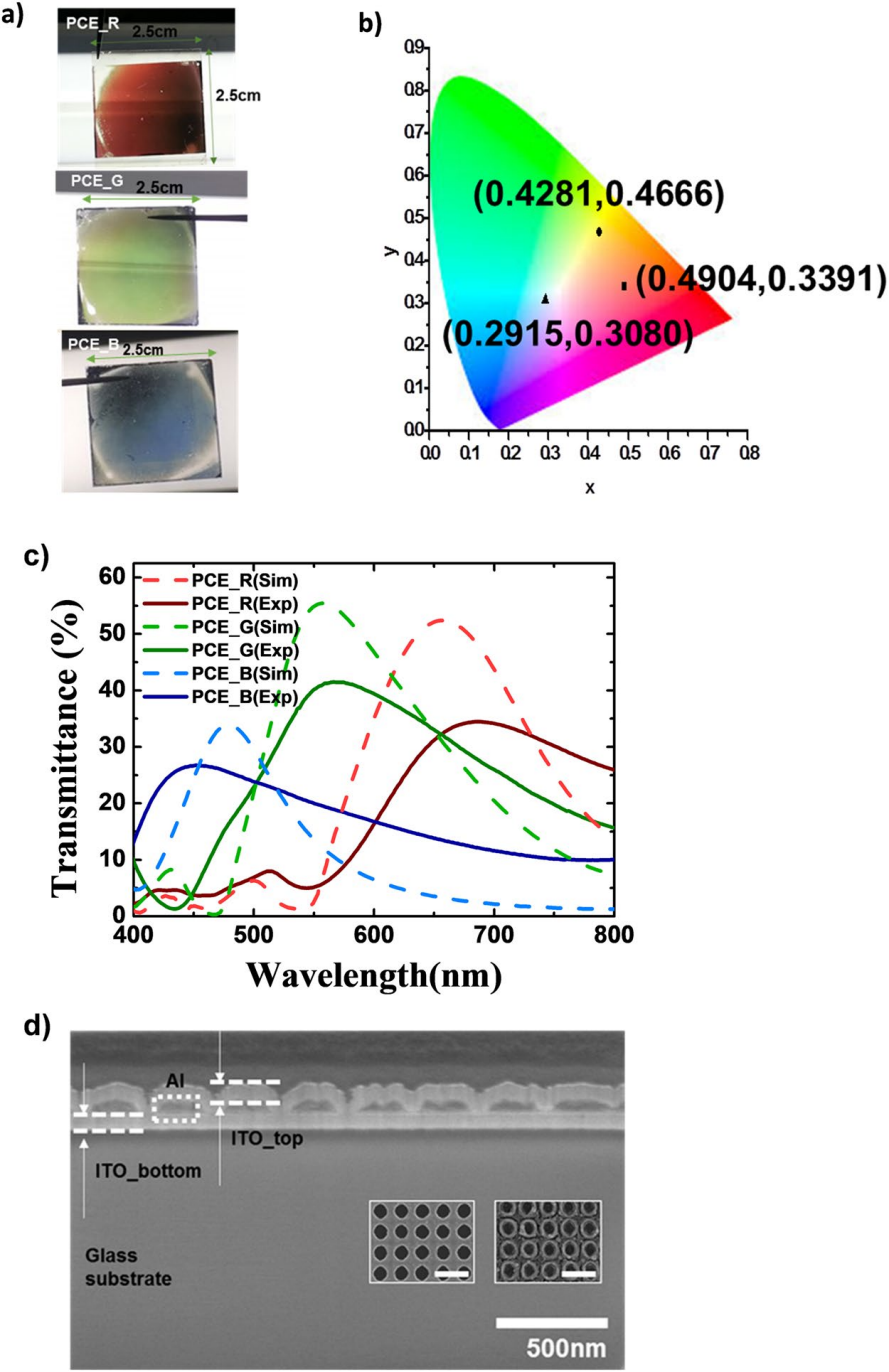
Optical characteristics of the fabricated PCE_R: (**a**) Photograph of the fabricated PCEs under white backlight. (**b**) CIE chromaticity diagram. The measured colors correspond to the following (x,y) points: (0.4904, 0.3391) for PCE_R, (0.4281, 0.4666) for PCE_G, and (0.2915, 0.3080) for PCE_B; (**c**) Transmittance spectra of the simulated and fabricated PCEs; (**d**) Focused-ion-beam scanning electron microscope (FIB-SEM) image. Insets show surface images of the photo-resist layer after the developing process (left) and a surface image of the Al layer after LIL (right). All of the scale bars in (**d**) are equal to 500 nm.

Figure 4 summarizes the results of the fabricated PCEs. As shown in Fig. 4a, the fabricated PCEs represented the colors red, green and blue, respectively. The color gamut was mapped on the CIE 1931 xy chromaticity diagram and the measured points for the PCE_R, PCE_G and PCE_B corresponded to the xy points of (0.4904, 0.3391), (0.4281, 0.4666) and (0.2915, 0.3080), respectively. Compared to the simulated results, the fabricated PCEs exhibited reduced performance. For PCE_R, the broader bandwidth resulted in an λ_SP_ at 677 nm. And the maximum transmittance obtained was 34.2%, dropping to 0.653 times as high as the calculated value, with more green and blue noise. PCE_G and PCE_B showed maximum transmittance of 41.5% at 567 nm and 28.1% at 449 nm, respectively, which are 0.760 times and 0.829 times lower than the calculation.

Degradation of the optical characteristics originated with imperfections due to fabrication. Unlike the assumptions in the simulation, the top surface of the PCE had some roughness, and the ITO layer did not perfectly cover the holes. Compared to the photoresist, following the LIL process debris was left around the edges of the holes on the surface of the Al hole layer, as shown in the insets of Fig. 4d. This produced an irregular top surface morphology, which consequently induced random scattering, which decreased EOT efficiency. And the laser exposoure in LIL was performed in non-ideal environment without controlling the air condition of contamination and vibration.

**Figure 5:**
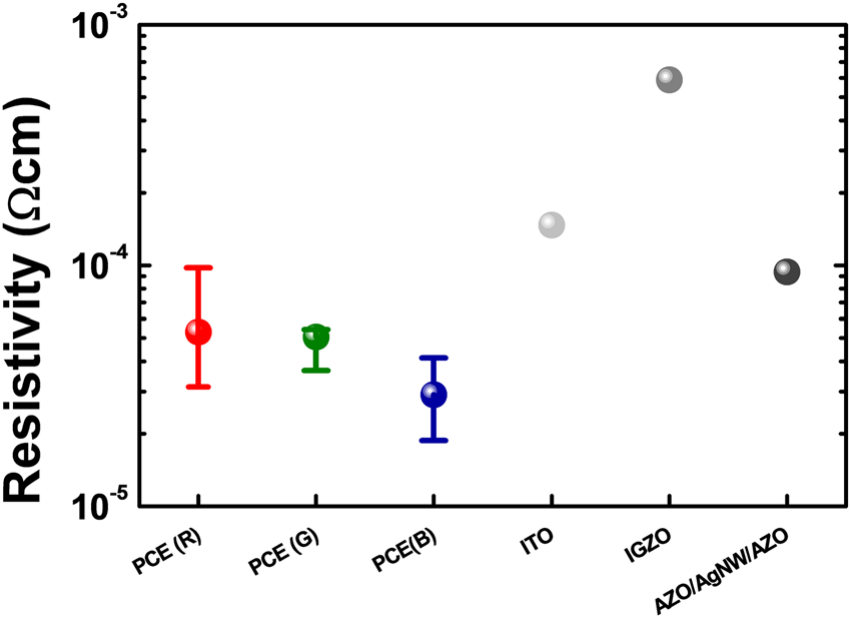
Electrical characteristic of the PCEs: Resistivity results of the PCEs compared to other reported transparent electrode materials.

We investigated the electric properties of the fabricated PCEs in comparison to conventional transparent electrode materials as shown in Fig. 5: i) Indium-gallium-zinc-oxide (IGZO);^29^ ii) a stacked layer consisting of Al doped zinc oxide (AZO) / AgNW / AZO layers in turn^30^. Considering the difference in thickness of each component, we compared electrical properties based on the resistivities caculated by the following:

2 $$\rho ={R}_{S}\times T$$

where ρ is the resistivity (Ωcm), R_S_ and T are the sheet resistance and the thickness of the electrode material, respectively. The measured R_S(PCE_R)_ was 1.42 Ω/sq with a thickness of 220 nm, which corresponds to 3.13 × 10^−5^ Ωcm of ρ_(PCE_R)_. For the same same thickness, PCE_G resulted in 1.92 Ω/sq, corresponding to 3.67 × 10^−5^ Ωcm of ρ_(PCE_G)_. In case of PCE_B, the measured R_S(PCE_B)_ was 1.38 Ω/sq with a thickness of 145 nm, which corresponds to 2.91 × 10^−5^ Ωcm of ρ_(PCE_B)_. For the 150 nm thick ITO layer in our experiment, R_S(ITO)_ was 9 Ω/sq and thus ρ_(ITO)_ was equal to 1.47 × 10^−4^ Ωcm. The reported values, R_S(IGZO)_ and R_S(AZO/AgNW/AZO)_, were 5.91 × 10^−4^ Ωcm and 9.4 × 10^−5^ Ωcm, respectively^29,30^.

The PCEs exhibited quite small resistivity. Even compared with conventional transparent electrode materials with ρ of roughly 10^−4^ Ωcm, our results show the proposed PCEs are likely to operate as good electrodes. The low resistivity comes from the high conductivity of the Al. Also, the continuous media of the ITO layers at the top and bottom compensates the loss of conductivity in the Al film, which has discontinuities due to the holes. Finally, this resulted in lower resistivity than a bare ITO layer.

## Discussion

Since plasmonic materials consist of metals, they are well suited for use as conducting materials. However previous attempts to deal with their color and electrically conducting features simultaneously have been less than successful. MNAs produce both SPs along the grating direction and LSPs around the holes, and therefore generate additional coupling chances between various light modes. That results in high transmittance at a resonance frequency which consequently produces better color selectivity as well as high transmittance. The combination of holes in the Al film and the continuous ITO media resulted in sufficiently low resistance for application as an electrode material.

For practical use of color filters, the angle response of a color is important. In periodic structures, the light modes participating in resonance have the momentums in the grating direction. Therefore PCEs in this work inevitably shows iridescent characteristics. In order to overcome this nature of periodic structures, we previously reported a poly-periodic hole array (PPHA) pattern which is consist of domains: holes are arranged periodically in one domain; each domain has different periodic axis; a unit domain is scaled in the propagation length of SPs^31^. The nano array in each domain works as an infinite array, and the disordered domain helps reducing color change in viewing angle.

Considering the absence of controlling dusts and vibration of the air in our experiment, there must be the improved filtering characteristics more coincident with the calculations. The finalized thickness of PCE_R, for example, was 220 nm. This thin and metal-based structure is advantageous for integrated on shape-free devices such as flexible, stretchable, and etc. Also, with the nano fabrication, PCE would realize much higher resolution than conventional color filters which have limitations on lithography of color resists.

## Conclusion

In conclusion, we propose a novel PCE to reduce the structural complexity of electric imaging devices. The thin layer functions as both a color filter and an electrode. The SP modes on the top and bottom interfaces match each other when the top dielectric layer fully fills the holes and creates a flat morphology. This leads to good color selectivity at the SPR wavelength compared to other nanostructure-based chromatic electrodes. Electrically, the fabricated PCE exhibited low resistivity values, measured at the level of 10^−5^ Ωcm, which is superior to conventional transparent electrodes. The results show the high feasibility of integrating nanostructures on conventional devices, and thus will provide additional opportunities for nano optical technologies to be employed in industrial applications.

## Method

### Numerical simulation

We performed the numerical analysis using the finite-different time-domain (FDTD) method (FDTD solutions, Lumerical). The unit cell was simulated using one periodicity with the assumption that the structure was infinitely periodic. This was possble because 2.5 cm is large enough to be compared with the SP propagation length. The boundary conditions were set to be anti-symmetric along the x axis and symmetric along the y axis, according to the direction of the electrical field, and pml was set along the z axis. The electric field propagated in the z direction from the bottom. The complex refractive index of ITO was measured with a spectroscopic ellipsometer (M2000D, Woollam). The refractive indexes of the Al and glass were as reported in the literature^32^.

### Fabrication

The glass was cleaned with acetone and isopropyl alcohol (IPA) by sonication for 10 min, respectively, and then treated by air plasma. A 75 nm ITO film was fabricated on 2.5 cm × 2.5 cm cleaned bare glass by RF-magnetron sputter. The post annealing process was performed in a 350 °C vacuum oven. The temperature was raised to 350 °C over a period of 2 h and kept at 350 °C for 2 h before falling to room temperature for 3 h. A 70 nm thick Al layer was evaporated on sputtered the 75 nm thick ITO glass by thermal evaporator. An adhesion layer was coated (HMDS:PGMEA = 1:4) before the photoresist (AR-N4240, mixed with thinner AR 300-12 at a ratio of 1:1) was spin-coated onto the sample, yielding a resist thickness of 300 nm. Then, a 2D hole array was patterned on the PR layer by laser interference lithography (LIL) with Lloyd’s mirror. After development, circular holes with a diameter of 180 nm were patterned every 300 nm^13^. After the LIL process, chlorine (Cl_2_) based inductive coupling plasma reactive ion etching (ICP-RIE, TCP-9600DFM, Lam Research) was conducted to etch the Al layer for 16 s. In order to strip the PR, the sample was immersed in acetone for 5 min, and then for 10 min in new acetone. After the stripping process, the sample was immersed in IPA for 10 min to clean. Then, PR residue was removed by air plasma (PDC-32G-2, Harrick Plasma). After the stripping process, 75 nm thick ITO was sputtered and post-annealed in the same way.

### Measurement

The sheet resistance of the PCEs was measured by four-point probe station (FFP-2400, Dasoleng). The transmittance spectra of the fabricated PCEs were measured with a UV-vis spectrophotometer (UV-2550, Shimadzu).

## Electronic supplementary material

Supporting Information

## References

[1] Chung K (2012). Flexible, angle-independent, structural color reflectors inspired by morpho butterfly wings. Adv. Mater..

[2] Aguirre CI, Reguera E, Stein A (2010). Tunable Colors in Opals and Inverse Opal Photonic Crystals. Adv. Funct. Mater..

[3] Choi H-JH (2013). Transmission-type photonic crystal structures for color filters. Opt. Express.

[4] Sabnis RW (1999). Color filter technology for liquid crystal displays. Displays.

[5] Wu YY-KR, Hollowell AEA, Zhang C, Guo LJ (2013). Angle-insensitive structural colours based on metallic nanocavities and coloured pixels beyond the diffraction limit. Sci. Rep..

[6] Xu T, Wu Y-K, Luo X, Guo LJ (2010). Plasmonic nanoresonators for high-resolution colour filtering and spectral imaging. Nature communications.

[7] Zhu P, Guo L, Jay Guo L (2012). High performance broadband absorber in the visible band by engineered dispersion and geometry of a metal-dielectric-metal stack. Appl. Phys. Lett..

[8] Han JH, Kim D-Y, Kim D, Choi KC (2016). Highly conductive and flexible color filter electrode using multilayer film structure. Sci. Rep..

[9] Barnes WLW, Dereux A, Ebbesen TWT (2003). Surface plasmon subwavelength optics. Nature.

[10] Genet C, Ebbesen TW (2007). Light in tiny holes. Nature.

[11] Park J, Kim M, Shin JB, Choi KC (2014). Transparent chromatic electrode using the mixture of silver nanowire and silver nanoprism. Curr. Appl. Phys..

[12] Do YS, Choi KC (2013). Matching Surface Plasmon Modes in Symmetry-Broken Structures for Nanohole-Based Color Filter. IEEE Photonics Technol. Lett..

[13] Do YS (2013). Plasmonic Color Filter and its Fabrication for Large-Area Applications. Adv. Opt. Mater..

[14] Chang S (2014). Photo-Insensitive Amorphous Oxide Thin-Film Transistor Integrated with a Plasmonic Filter for Transparent Electronics. Adv. Funct. Mater..

[15] Bethe HA (1944). Theory of Diffraction by Small Holes. Phys. Rev..

[16] Lalanne P, Hugonin JP (2006). Interaction between optical nano-objects at metallo-dielectric interfaces. Nat. Phys..

[17] van Beijnum F (2012). Quasi-cylindrical wave contribution in experiments on extraordinary optical transmission. Nature.

[18] Degiron A, Ebbesen TW (2005). The role of localized surface plasmon modes in the enhanced transmission of periodic subwavelength apertures. J. Opt. A Pure Appl. Opt..

[19] Martín-Moreno L (2001). Theory of Extraordinary Optical Transmission through Subwavelength Hole Arrays. Phys. Rev. Lett..

[20] Ebbesen T, Lezec H, Ghaemi H, Thio T, Wolff P (1998). Extraordinary optical transmission through sub-wavelength hole arrays. Nature.

[21] Hu X, Zhan L, Xia Y (2008). Color filters based on enhanced optical transmission of subwavelength-structured metallic film for multicolor organic light-emitting diode display. Appl. Opt..

[22] Park TT-H (2008). Optical properties of a nanosized hole in a thin metallic film. ACS Nano.

[23] Knight MW (2014). Aluminum for plasmonics. ACS Nano.

[24] Naik GV, Shalaev VM, Boltasseva A (2013). Alternative plasmonic materials: beyond gold and silver. Adv. Mater..

[25] Liu H, Lalanne P (2008). Microscopic theory of the extraordinary optical transmission. Nature.

[26] Wolferen, H., Abelmann, L. & van Wolferen, H. in *Lithography: Principles*, Processes *and Materials* (ed. Hennessy, T. C.) 133–148, at http://doc.utwente.nl/78097/ (Nova Science Publishers, Inc., 2011).

[27] Xie Q (2008). Fabrication of nanostructures with laser interference lithography. J. Alloys Compd..

[28] Lu C, Lipson RH (2009). Interference lithography: a powerful tool for fabricating periodic structures. Laser Photon. Rev..

[29] Hsu C-M, Tzou W-C, Yang C-F, Liou Y-J (2015). Investigation of the High Mobility IGZO Thin Films by Using Co-Sputtering Method. Materials (Basel)..

[30] Huang Q (2015). Highly thermostable, flexible, transparent, and conductive films on polyimide substrate with an AZO/AgNW/AZO structure. ACS Appl. Mater. Interfaces.

[31] Do YS, Choi KC (2015). Poly-periodic hole arrays for angle-invariant plasmonic filters. Opt. Lett..

[32] Handbook of Optical Co*nstants of Solids*. *Handbook of Optical Constants of Solids*, 10.1016/B978-012544415-6.50000-5 (Elsevier, 1997).

