# Author Correction: Plasmonic Chromatic Electrode with Low Resistivity

**DOI:** 10.1038/s41598-018-28933-6

**Published:** 2018-07-11

**Authors:** Young Gyu Moon, Yun Seon Do, Min Ho Lee, Bo Yeon Hwang, Dong Jun Jeong, Byeong-Kwon Ju, Kyung Cheol Choi

**Affiliations:** 1 Korea Advanced Institute of Science and Technology (KAIST), School of Electrical Engineering, Daejeon, 34141 Republic of Korea; 2 Kyungpook National University, School of Electronics Engineering, Daegu, 41566 Republic of Korea; 3 Korea University, School of Electrical Engineering, Seoul, 136-713 Republic of Korea

Correction to: *Scientific Reports* 10.1038/s41598-017-15465-8, published online 09 November 2017

The original version of this Article contained an error in Affiliation 2, which was incorrectly given as ‘Kyungpook National University, School of Electrical Engineering, Daegu, 41566, Republic of Korea’. The correct affiliation is listed below:

Kyungpook National University, School of Electronics Engineering, Daegu, 41566, Republic of Korea

This error has now been corrected in the HTML and PDF versions of the Article.

