# Supplementary material for: Plasmonic Chromatic Electrode with Low Resistivity
Source: Sci Rep. 2017 Nov 9;7:15206. doi: 10.1038/s41598-017-15465-8 (PMC5680321; doi:10.1038/s41598-017-15465-8)
Supplement: Supplementary file 1 — Supporting Information [file 41598_2017_15465_MOESM1_ESM.pdf]

Supporting Information

Plasmonic chromatic electrode with low resistivity

Young Gyu Moon[1†], Yun Seon Do[2†], Min Ho Lee[1†], Bo Yeon Hwang[3], Dong Jun Jeong[1], Byeong-Kwon Ju[3] and Kyung Cheol Choi[1*]*

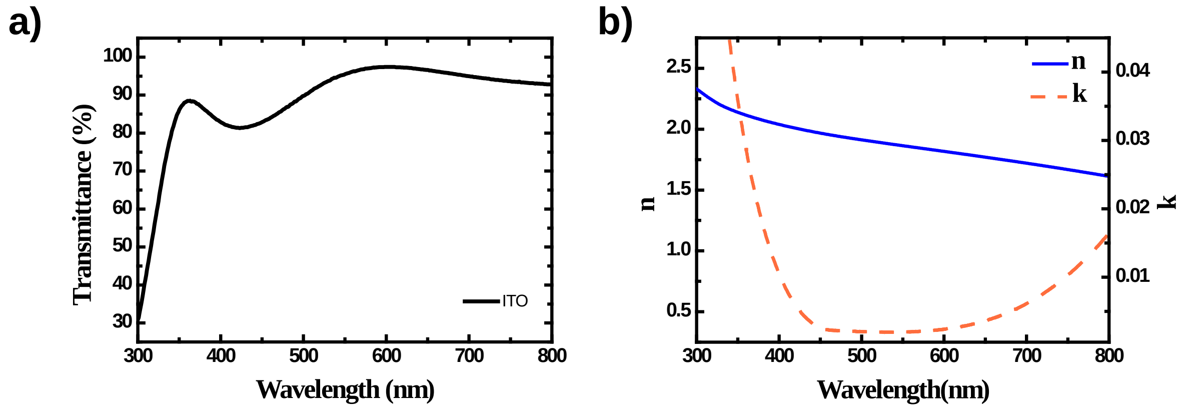

Figure S1. The optical characteristic of ITO: a) Transmission spectrum of ITO. b) Complex refractive index of ITO from the ellipsometer.

Figure S1a and S1b show actual values obtained from measuring equipment (UV spectrometer (UV-2550, Shimadzu), ellipsometer (M-2000D, Woollam)). Figure S1b indicates complex refractive index of ITO. According to figureS1b, the refractive index of ITO was 1.7 to 2.4 in visible range, so large refractive index difference between ITO and glass caused light loss in ITO layer. Also the extinction coefficient denoted by κ of ITO was not negligible. In the visible range, therefore, the optical loss in ITO layer is occurred.

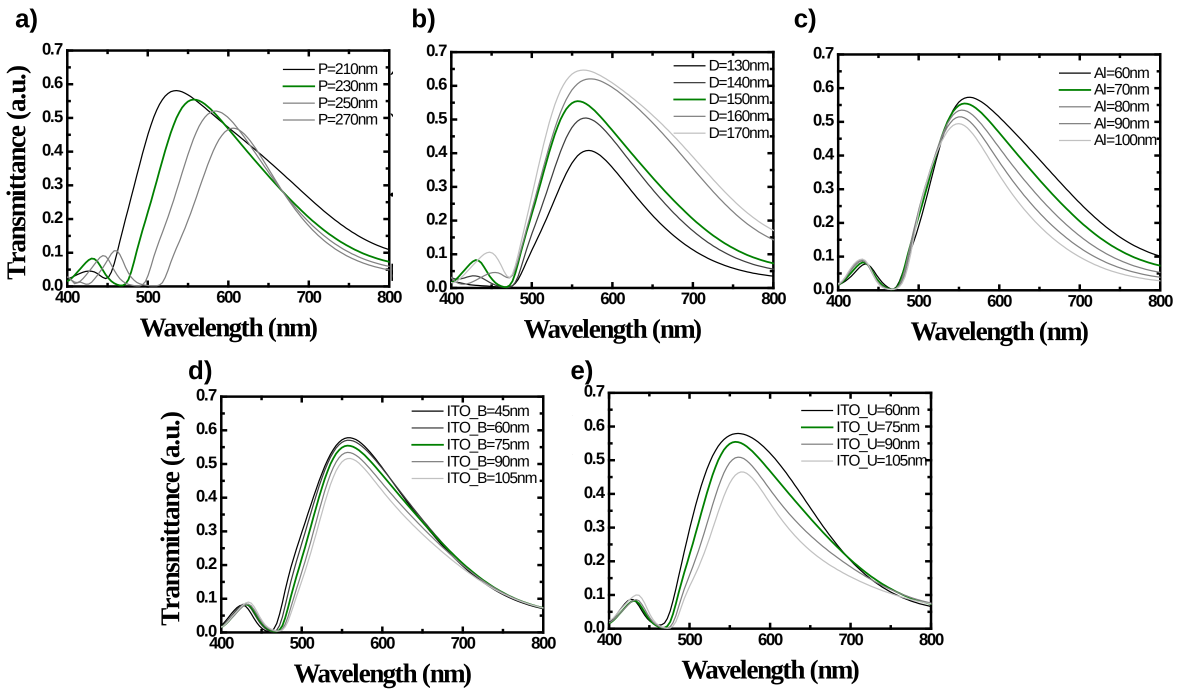

Figure S2. Simulated transmission responses to design the green PCE according to a) period; b) diameter; c) thickness of Al; d) thickness of ITO_bottom; e) thickness of ITO_top.

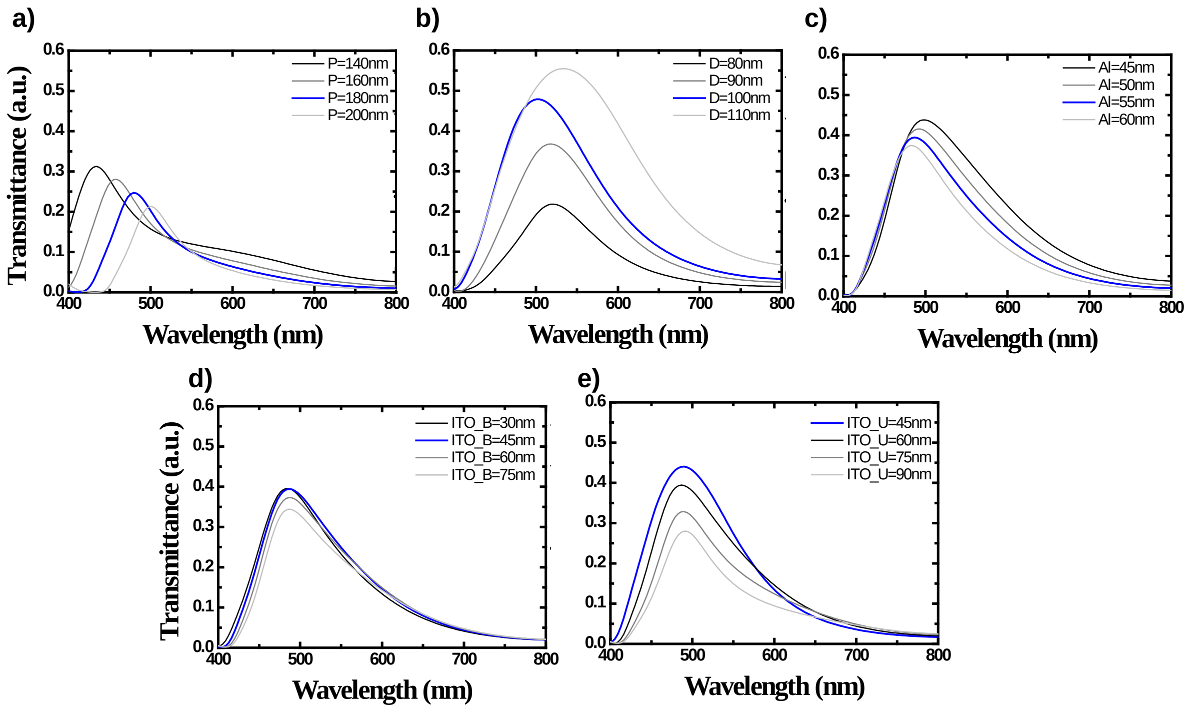

Figure S3. Simulated transmission responses to design the blue PCE according to a) period; b) diameter; c) thickness of Al; d) thickness of ITO_bottom; e) thickness of ITO_top.

Figure S2 and Figure S3 show transmission spectra of MNAs, designed for passing green and blue color region according to varying parameters. We designed electrodes in order of Figure S2 and Figure S3: a) period of MNAs, P; b) diameter of holes, D; c) thickness of the Al film, T; d) thickness of the bottom ITO layer, ITO_B; e) thickness of the top ITO layer, ITO_U. Following table represents the information, varying range of a parameter and fixed dimensions for calculating each green spectrum. In line with designing for red electrode (Figure 3), except for a determining dimension, we set the other parameters as initial values or concluded values.

Design factors (green)	Period (P)	Diameter (D)	Al Thickness (T)	ITO_B Thickness	ITO_U Thickness
P_sweep	210-280	150	70	75	75
D_sweep	230	100-190	70	75	75
Al_sweep	230	150	55-100	75	75
ITO_B_sweep	230	150	70	15-150	75
ITO_U_sweep	230	150	70	75	60-150

Following table represents the information, varying range of a parameter and fixed dimensions for calculating each blue spectrum. In line with designing for red electrode (Figure 3), except for a determining dimension, we set the other parameters as initial values or concluded values.

Design factors (blue)	Period (P)	Diameter (D)	Al Thickness (T)	ITO_B Thickness	ITO_U Thickness
P_sweep	160-190	100	45	45	45
D_sweep	180	80-130	45	45	45
Al_sweep	180	100	35-60	45	45
ITO_B_sweep	180	100	55	0-90	45
ITO_U_sweep	180	100	55	45	30-55

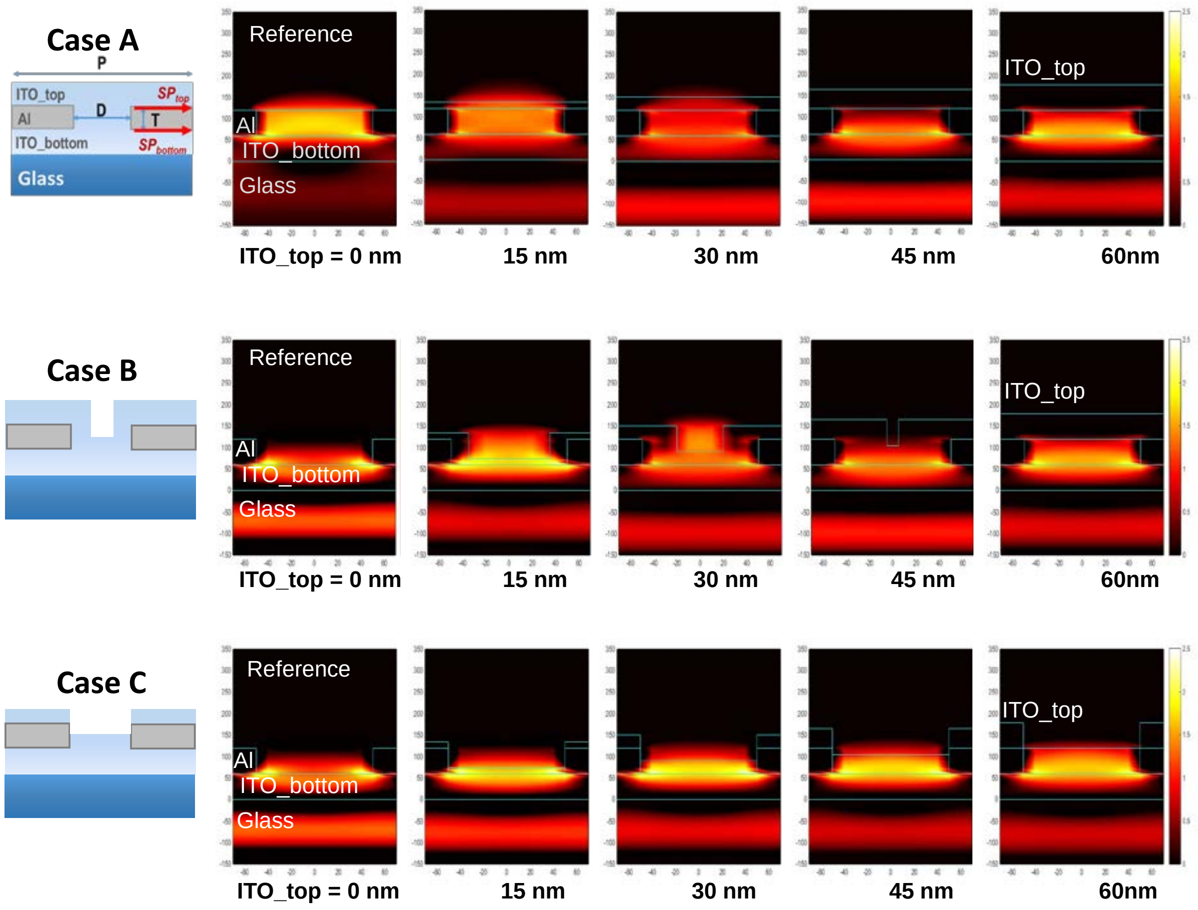

Figure S4. Electric field profiles at the wavelength of maximum transmittance.

Figure S4 shows the electric field (E-field) profiles of each structure according to the thickness of ITO_top layer. In Case A and Case B, thin ITO overlayer (~30 nm), E-field enhancement around the hole was shown in both up and bottom side. However, in Case C, E-field was strongly confined only at the bottom side of the hole. This is in accordance with our interpretation of SP matching condition in the manuscript.
